# Association of Diet Quality, Dietary Acid Load, and Dietary Antioxidant Index With Cardiometabolic and NAFLD Risk Factors Among Patients With Metabolic Syndrome: A Cross‐Sectional Study

**DOI:** 10.1002/fsn3.71102

**Published:** 2025-10-17

**Authors:** Bijan Ghobadian, Seyedeh Atiye Shahrokhi, Saman Rezaei Talabon, Vahid Notash, Fatemeh Maleki Sedgi, Mehran Rahimlou

**Affiliations:** ^1^ Metabolic Diseases Research Center, Health and Metabolic Research Institute Zanjan University of Medical Science Zanjan Iran; ^2^ Department of Nutrition, School of Public Health Zanjan University of Medical Sciences Zanjan Iran

**Keywords:** diet quality, dietary acid load, metabolic syndrome, non‐alcoholic fatty liver disease, potential renal acid load

## Abstract

Metabolic syndrome (MetS) is closely associated with non‐alcoholic fatty liver disease (NAFLD). Diet quality, acid–base balance, and antioxidant intake may influence NAFLD risk. This study examines the relationship between the Diet Quality Index (AHEI), Dietary Acid Load (DAL), and Dietary Antioxidant Index (DAI) with liver enzyme levels, lipid profiles, and NAFLD predictors in individuals with MetS. A cross‐sectional study was conducted on 420 adults diagnosed with MetS according to International Diabetes Federation criteria in Zanjan, Iran. Dietary intake was assessed using a validated food frequency questionnaire. AHEI, DAL, and DAI were calculated. Liver enzymes, lipid profiles, and predictive indices, including the Fatty Liver Index (FLI), Hepatic Steatosis Index (HSI), and Triglyceride‐Glucose (TyG) Index, were analyzed. Multivariable regression adjusted for confounders. Higher AHEI (tertile 3 vs. tertile 1) was associated with lower FLI (65.37 ± 10.46 vs. 74.37 ± 11.39 (all values presented as mean ± standard deviation [SD]); *p* < 0.001), HSI (40.49 ± 6.17 vs. 47.28 ± 7.19; *p* = 0.02), triglycerides (163.40 ± 62.19 mg/dL vs. 174.43 ± 63.72 mg/dL; *p* = 0.034), ALT (22.67 ± 6.29 IU/L vs. 29.39 ± 7.43 IU/L; *p* = 0.023), and GGT (20.37 ± 7.12 IU/L vs. 27.91 ± 8.43 IU/L; *p* = 0.033), and higher HDL cholesterol (49.23 ± 15.93 mg/dL vs. 43.24 ± 13.39 mg/dL; *p* = 0.042). Higher DAL (tertile 3 vs. tertile 1) was linked to elevated FLI (79.43 ± 12.13 vs. 69.33 ± 10.36; *p* < 0.001) and ALT (30.75 ± 8.23 IU/L vs. 19.76 ± 5.43 IU/L; *p* = 0.002). Higher DAI (tertile 3 vs. tertile 1) was inversely associated with HSI (41.8 ± 5.1 vs. 46.5 ± 6.7; *p* = 0.005). Higher AHEI and DAI scores were associated with better liver function and lipid profiles, while higher DAL correlated with worse outcomes. Improving diet quality and balancing acid–base intake may be associated with reduced NAFLD risk in MetS patients.

## Graphical abstract

This cross‐sectional study of 420 patients with metabolic syndrome found that higher Alternative Healthy Eating Index (AHEI) and Dietary Antioxidant Index (DAI) scores were associated with improved lipid profiles, lower liver enzymes, and reduced non‐alcoholic fatty liver disease (NAFLD) risk indices. Conversely, higher Dietary Acid Load (DAL) was linked to elevated liver enzymes and increased NAFLD risk. These findings highlight the potential role of diet quality, acid–base balance, and antioxidant intake in mitigating NAFLD risk.

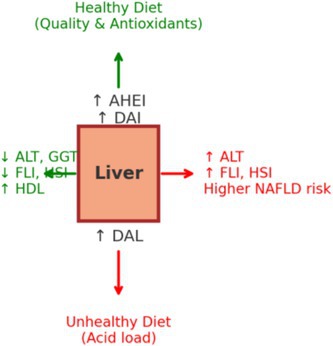

## 1 Introduction

Metabolic dysfunction–associated steatotic liver disease (MASLD), formerly known as nonalcoholic fatty liver disease (NAFLD), was redefined following the 2023 international multisociety consensus and further highlighted in the 2024 EASL–EASD–EASO clinical practice guidelines (Huang et al. 2025; Radu et al. 2023). This change emphasizes the metabolic underpinnings of the disease and provides a unified framework for both research and clinical management. According to recent global estimates, MASLD affects approximately 30%–38% of the adult population and is projected to exceed 40% by 2050, underscoring the urgent need for preventive strategies (Younossi et al. 2024). MASLD is strongly associated with MetS components, including hypertension, dyslipidemia (elevated triglycerides and low levels of high‐density lipoprotein [HDL] cholesterol), hyperglycemia, and central obesity (Driessen et al. 2025).

Emerging evidence suggests that MetS is strongly linked to insulin resistance, a key factor in the pathogenesis of NAFLD (Grander et al. 2016; Portincasa et al. 2024). NAFLD is characterized by excessive fat accumulation in hepatocytes in the absence of significant alcohol consumption or other secondary causes of hepatic steatosis (Yki‐Järvinen 2014). Recent studies estimate the global prevalence of NAFLD at 32.4%, with an Iranian study in 2023 reporting a prevalence of approximately 33% (Radu et al. 2023; Tabaeian et al. 2024). Notably, not all individuals with MetS develop NAFLD, and not all individuals with NAFLD progress to non‐alcoholic steatohepatitis (NASH) or advanced liver disease (Browning et al. 2004).

Dietary patterns have increasingly been recognized as central determinants of MASLD risk (Li et al. 2024). Recent studies (2023–2025) have shown that higher scores on diet quality indices such as the Healthy Eating Index (HEI) and the Alternative Healthy Eating Index (AHEI) are associated with lower odds of MASLD and reduced severity of steatosis and fibrosis (Zhang et al. 2022).

Diet quality is typically assessed using dietary indices that quantify adherence to nutritional guidelines and patterns associated with health outcomes (Lupton et al. 2016). We selected the Alternative Healthy Eating Index (AHEI) over alternatives like HEI‐2025 due to its emphasis on specific components highly relevant to NAFLD pathogenesis, including polyunsaturated fats, nuts and legumes, and limitations on processed meats and trans fats, which align closely with metabolic and hepatic outcomes (Onvani et al. 2017). In contrast, HEI‐2025, while comprehensive in assessing overall adherence to the Dietary Guidelines for Americans (2025–2030), places a more balanced emphasis on broader categories such as total protein foods, refined grains, and added sugars, without the same prioritization of NAFLD‐specific protective elements like the PUFA‐to‐SFA ratio or moderate alcohol intake. A higher AHEI score has been linked to improved metabolic health, reduced inflammation, and lower risks of chronic diseases, including NAFLD (Marshall et al. 2014).

Beyond overall diet quality, DAL has garnered attention as a potential contributor to metabolic dysregulation. DAL reflects the balance between acid‐forming and base‐forming dietary components. Diets high in acidogenic foods (e.g., animal proteins, processed grains, and dairy) and low in alkaline foods (e.g., fruits and vegetables) may promote metabolic acidosis, insulin resistance, and hepatic lipid accumulation (Engberink et al. 2012; van den Berg et al. 2011). Previous research has indicated that higher DAL is associated with unfavorable metabolic outcomes, including hypertension, insulin resistance, and an increased risk of NAFLD (Reddy et al. 2002; Sakhaee et al. 2002).

Furthermore, oxidative stress is a well‐established driver of metabolic dysfunction and liver disease progression. DAI is used to evaluate the cumulative antioxidant potential of dietary intake, incorporating key micronutrients such as vitamins A, C, and E, selenium, manganese, and zinc (Liu et al. 2015; Rives et al. 2020). Antioxidants play a pivotal role in neutralizing reactive oxygen species (ROS), mitigating oxidative damage, and modulating inflammatory pathways (Wang and Yi 2022). While individual antioxidants have been investigated for their potential benefits in NAFLD management, the collective impact of dietary antioxidants, as measured by DAI, remains an area of active research.

Given these considerations, the present study aims to investigate the associations between AHEI, DAL, and DAI with key metabolic and hepatic biomarkers in patients with MetS. Specifically, we examine their relationships with liver enzyme levels, lipid profiles, and established NAFLD risk indices, including the FLI, HSI, and TyG Index. We hypothesized that higher AHEI and DAI scores would be inversely associated with NAFLD risk indices and biomarkers, while higher DAL would be positively associated with these outcomes. Understanding these associations may provide valuable insights for dietary interventions targeting NAFLD prevention and management in high‐risk populations.

## 2 Methods

### 2.1 Design of the Research Study and Gathering of Samples

The study followed the guidelines outlined in the Declaration of Helsinki (Carlson et al. 2004), and all procedures that involved human subjects or patients were approved by the Ethics Committee of Zanjan University of Medical Sciences (Ethics Code: IR.ZUMS.REC.1403.004). All participants provided written and informed consent, and this was obtained from all subjects/patients. The study included patients diagnosed with MetS who attended the outpatient endocrine clinic of Vali‐e‐Asr Hospital and a specialized endocrinology practice in Zanjan, Iran, in 2024.

Based on the standard deviation (SD) of dietary diversity index (5.38) reported by Taghdir et al. (2023), a sample size of 380 participants was calculated using a 95% confidence interval and assuming a type I error of 0.05 and power of 80%. This SD was selected as a proxy for dietary index variability due to the lack of prior data on AHEI, DAL, or DAI in Iranian MetS patients; dietary diversity correlates strongly with overall diet quality in similar populations. Considering potential dropouts, the final sample size was increased to 420 participants. Participants were recruited using simple random sampling from eligible individuals visiting the specified clinics.

### 2.2 Inclusion and Exclusion Criteria

Inclusion criteria for the study included individuals diagnosed with MetS based on the International Diabetes Federation (IDF) criteria. This required a waist circumference of at least 94 cm for men or 80 cm for women, along with the presence of at least two of the following factors: systolic blood pressure of 130 mmHg or higher, diastolic blood pressure of 85 mmHg or higher; fasting blood glucose levels of 100 mg/dL or higher; triglyceride levels of 150 mg/dL or higher; and HDL cholesterol levels below 40 mg/dL for men or below 50 mg/dL for women (Alberti et al. 2005). Additional inclusion criteria specified that participants should not have used antioxidant supplements (such as vitamin E, selenium, or omega‐3) or medications and supplements affecting liver fat (such as metformin, Liv‐52, or curcumin) in the past 3 months. Furthermore, participants should not have followed restrictive diets (such as very low‐calorie, ketogenic, or vegetarian diets) in the past 3 months. This exclusion of recent antioxidant supplement use was implemented to minimize confounding in the calculation and analysis of the DAI, which is based exclusively on dietary intake, ensuring that associations with metabolic and NAFLD risk factors reflect food‐derived antioxidants rather than supplemental sources (e.g., vitamin C or E).

Exclusion criteria included a lack of consent to participate, pregnancy or lactation, the presence of autoimmune diseases or cancer, an average daily caloric intake below 800 kcal or above 4500 kcal, and the presence of liver cirrhosis, fibrosis, or hepatocellular carcinoma.

### 2.3 Data Collection and Measurements

Dietary intake over the past year was assessed using a validated 168‐item food frequency questionnaire (FFQ) administered by trained dietitians (Esfahani et al. 2010). Nutrient and dietary index calculations were performed using N4 software.

### 2.4 Anthropometric and Clinical Data

Weight, height, and waist circumference were measured using standard protocols. Body mass index (BMI) was calculated as weight (kg) divided by height squared (m^2^). Laboratory assessments included fasting blood glucose, lipid profile (triglycerides, LDL, HDL, total cholesterol), and liver enzymes (ALT, AST, GGT). Type 2 diabetes status for the HSI calculation was determined based on fasting blood glucose ≥ 126 mg/dL or use of antidiabetic medications, assessed separately from MetS hyperglycemia criteria.

## 3 Dietary Indices Calculation

### 3.1 AHEI

This index includes 11 components, with higher scores indicating healthier diets. Positive components include legumes, vegetables, whole grains, nuts, fruits, DHA/EPA, and polyunsaturated fats. Negative components include trans fats, sweetened beverages, sodium, and processed/red meats. Alcohol consumption is scored neutrally. Scores ranged from 9 to 81, with higher scores reflecting better diet quality.

### 3.2 DAL

DAL was calculated using two methods (Han et al. 2016):

PRAL (mEq/day) = 0.4888 × dietary protein (g/day) + 0.0366 × dietary phosphorus (mg/day) − 0.0205 × dietary potassium (mg/day) − 0.0125 × dietary calcium (mg/day) − 0.0263 × dietary magnesium (mg/day). The second method calculated DAL using body surface area (BSA) and PRAL:

$$\begin{array}{c} \mathrm{DAL}\;\left(\mathrm{mEq}/\mathrm{day}\right)=\left(\text{body surface area}\;\left[m^{2}\right]\times 41\;\left[\mathrm{mEq}/\mathrm{day}\right]/1.73\;m^{2}\right)\\ +\text{PRAL} \end{array}$$

where body surface area was calculated using the Du Bois formula: height^0.725^ × weight^0.425^ × 0.00718.

### 3.3 DAI

The DAI was calculated based on intake of vitamin A, C, E, selenium, manganese, and zinc. Standardized scores were adjusted for global means and standard deviations and subsequently normalized for energy intake (LuuHung 2015). Global means and standard deviations were derived from international reference data (e.g., global and United States intake estimates) as reported in previous studies (LuuHung 2015).

$$\mathrm{DAI}=\sum_{i=1}^{n=6}\frac{\text{Individual intake}-\text{Mean}}{\mathrm{SD}}$$

### 3.4 NAFLD Prediction Indices

Three indices were calculated:

**FLI =** (e0.953 × Ln (triglycerides) + 0.139 × BMI + 0.718 × Ln (GGT) + 0.053 × WC − 15.745)/(1 + e0.953 × Ln (triglycerides) + 0.139 × BMI + 0.718 × Ln (GGT) + 0.053 × WC − 15.745) × 100 (Bedogni et al. 2006).

**TyG Index =** Ln (triglycerides [mg/dL] × glucose [mg/dL]/2) (Simental‐Mendía et al. 2008).

**HSI =** 8 × (ALT/AST) + BMI + 2 (if type 2 diabetes) + 2 (if female).

Risk categories for each index were used for analysis.

### 3.5 Statistical Analysis

Data were analyzed using SPSS version 26. Descriptive statistics included mean ± SD for continuous variables and frequencies for categorical variables. ANOVA was used to compare biochemical variables across dietary index quartiles. Chi‐square tests were employed for categorical comparisons. Linear and multiple regression analyses assessed relationships between dietary indices and NAFLD predictors, adjusting for potential confounders (age, sex, BMI, total energy intake, and percentages of energy from carbohydrates, proteins, and fats). Multicollinearity was assessed using variance inflation factors (VIF), with all values < 5 indicating no significant issues. No separate power analyses were conducted for subgroups, as the study was powered for primary outcomes; post hoc calculations confirmed > 80% power for key associations (e.g., AHEI with FLI). For FFQ data, participants with > 10% missing items were excluded; minor missing data were imputed using group means. Energy intake outliers were already excluded per study criteria. Statistical significance was set at *p* < 0.05.

## 4 Results

### 4.1 Demographic and Clinical Characteristics of Participants

**FIGURE 1:**
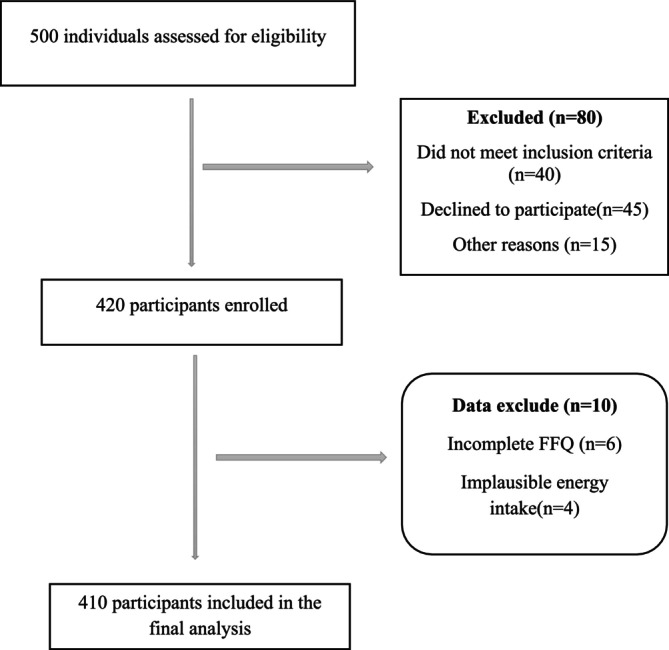
Flowchart of participant recruitment and inclusion in the study.

A total of 500 individuals were initially assessed for eligibility. Of these, 80 were excluded due to not meeting inclusion criteria (*n* = 40), declining participation (*n* = 25), or other reasons (*n* = 15). Ultimately, 420 participants were enrolled in the study. During data cleaning, 10 participants were excluded due to incomplete FFQ responses (*n* = 6) or implausible energy intake (< 800 or > 4500 kcal/day) (*n* = 4). Thus, 410 participants were included in the final analysis (Figure 1). The demographic and anthropometric characteristics of the study participants, stratified by tertiles of the Alternate Healthy Eating Index (AHEI), are presented in Table 1.

**TABLE 1:** Demographic, clinical, and anthropometric characteristics of study participants based on tertiles of the diet quality index.

Variables	Tertile 1 (*n* = 140) Mean ± SD *N* (%)	Tertile 2 (*n* = 140) Mean ± SD *N* (%)	Tertile 3 (*n* = 140) Mean ± SD *N* (%)	*p*
Age (years)	53.69 ± 10.26	51.37 ± 8.78	55.71 ± 11.32	0.07
Gender (Male)	45 (32.14%)	39 (27.85%)	43 (30.71%)	0.47
Marital Status (Married)	123 (87.85%)	118 (84.28%)	127 (90.71%)	0.53
Education Level				0.76
Below Diploma or Diploma	89 (63.57%)	85 (60.71%)	87 (62.14%)	
Bachelor's Degree	38 (27.14%)	43 (30.71%)	40 (28.57%)	
Master's or Doctorate	13 (9.29%)	12 (8.58%)	13 (9.29%)	
Weight (kg)	76.35 ± 8.43	79.61 ± 9.22	75.41 ± 8.36	0.59
BMI (kg/m^2^)	27.41 ± 5.34	28.36 ± 7.91	27.76 ± 6.37	0.55
Waist Circumference (cm)	94.27 ± 17.63	96.75 ± 20.15	94.69 ± 18.40	0.61
Fatty Liver Index (FLI)	74.37 ± 11.39	70.18 ± 13.45	65.37 ± 10.46	< 0.001
Hepatic Steatosis Index (HSI)	47.28 ± 7.19	45.34 ± 6.89	40.49 ± 6.17	0.02
TyG Index	7.23 ± 1.74	7.11 ± 1.63	6.96 ± 1.58	0.17
Fasting Blood Glucose (mg/dL)	94.17 ± 14.37	91.25 ± 15.29	93.76 ± 13.56	0.45
Triglycerides (mg/dL)	174.43 ± 63.72	169.59 ± 60.36	163.40 ± 62.19	0.034
Total Cholesterol (mg/dL)	189.43 ± 56.19	183.25 ± 50.41	185.37 ± 59.43	0.36
LDL Cholesterol (mg/dL)	116.46 ± 36.43	114.29 ± 38.25	115.28 ± 35.48	0.78
HDL Cholesterol (mg/dL)	43.24 ± 13.39	45.29 ± 15.26	49.23 ± 15.93	0.042
Alanine Aminotransferase (mg/dL)	29.39 ± 7.43	25.39 ± 5.44	22.67 ± 6.29	0.023
Aspartate Aminotransferase (mg/dL)	24.17 ± 5.48	23.84 ± 5.74	21.64 ± 4.85	0.37
Gamma‐Glutamyl Transferase (GGT)	27.91 ± 8.43	25.85 ± 7.26	20.37 ± 7.12	0.033
Systolic Blood Pressure (mmHg)	120.15 ± 8.35	118.43 ± 8.13	119.25 ± 7.90	0.71
Diastolic Blood Pressure (mmHg)	81.73 ± 12.86	81.59 ± 13.40	80.43 ± 11.76	0.37

*Note:* Data are presented as mean ± standard deviation (SD) for continuous variables and number (percentage) for categorical variables.

The mean age of participants in the first tertile of AHEI was 53.69 ± 10.26 years, while in the third tertile, it was 55.71 ± 11.32 years, with no statistically significant difference (*p* = 0.07). Of all participants, 30.23% were male, and the gender distribution differed significantly across tertiles.

The mean weight of participants in the first tertile of AHEI was 76.35 ± 8.43 kg, compared to 75.41 ± 8.36 kg in the third tertile, with no significant difference (*p* = 0.59). Similarly, no significant differences were observed in waist circumference and BMI across AHEI tertiles.

The results indicated that the mean Fatty Liver Index (FLI) score was significantly lower in the third tertile (65.37 ± 10.46) compared to the first tertile (74.37 ± 11.39, *p* < 0.001). Regarding the Hepatic Steatosis Index (HSI), participants in the third tertile also had significantly lower scores than those in the first tertile (*p* < 0.05).

Clinically, participants in the third tertile of AHEI had significantly lower mean levels of triglycerides (163.40 ± 62.19 mg/dL vs. 174.43 ± 63.72 mg/dL, *p* = 0.034), gamma‐glutamyl transferase (GGT) (20.37 ± 7.12 U/L vs. 27.91 ± 8.43 U/L, *p* = 0.033), and alanine aminotransferase (ALT) (22.67 ± 6.29 U/L vs. 29.39 ± 7.43 U/L, *p* = 0.023). They also had higher levels of HDL cholesterol (49.23 ± 15.93 mg/dL vs. 43.24 ± 13.39 mg/dL, *p* = 0.042).

### 4.2 Nutritional Intake Across AHEI Tertiles

The distribution of macronutrient and micronutrient intakes across AHEI tertiles is summarized in Table 2. The mean energy intake in the first tertile was 2565.77 ± 576.65 kcal/day, compared to 2737.45 ± 695.24 kcal/day in the third tertile, with no significant difference (*p* = 0.27). Similarly, no significant differences were observed in the intake of protein (*p* = 0.38), carbohydrates (*p* = 0.68), and fat (*p* = 0.34).

**TABLE 2:** Distribution of macronutrients, micronutrients, and predictors of fatty liver across different tertiles of the alternate healthy eating index (AHEI).

Variable	Tertile 1 (*n* = 140)	Tertile 2 (*n* = 140)	Tertile 3 (*n* = 140)	p*
AHEI	43.26 ± 7.12	59.48 ± 5.76	68.29 ± 5.74	< 0.001
Energy intake (kcal/day)	2565.77 ± 576.65	2675.19 ± 655.49	2737.45 ± 695.24	0.27
Protein (% of energy)	13.83 ± 5.7	14.12 ± 4.42	13.92 ± 3.95	0.38
Carbohydrate (% of energy)	56.54 ± 6.85	57.22 ± 7.23	56.87 ± 6.96	0.68
Fat (% of energy)	30.38 ± 5.83	31.45 ± 6.27	31.76 ± 6.12	0.34
Fiber (g/1000 kcal)	16.21 ± 7.73	16.54 ± 8.43	16.89 ± 7.69	0.29
Vitamin D (μg/day)	1.83 ± 0.68	2.31 ± 0.85	2.89 ± 1.22	< 0.001
Vitamin E (mg/day)	13.76 ± 8.25	16.51 ± 7.69	18.39 ± 8.43	0.03
Vitamin C (mg/day)	156.32 ± 86.73	181.43 ± 82.55	216.64 ± 93.46	< 0.001
Vitamin B6 (mg/day)	2.32 ± 0.57	2.59 ± 0.71	2.96 ± 0.68	0.037
Vitamin B9 (μg/day)	563.44 ± 219.35	574.29 ± 247.69	587.43 ± 236.37	0.23
Vitamin B12 (μg/day)	6.65 ± 2.85	7.19 ± 3.26	6.69 ± 2.95	0.15
Zinc (mg/day)	15.64 ± 6.37	17.48 ± 6.15	19.67 ± 7.36	< 0.001
Calcium (mg/day)	1423.16 ± 486.28	1578.26 ± 517.45	1734.46 ± 689.41	0.002
Vegetables (servings/day)	1.98 (1.12–2.85)	2.34 (1.87–3.56)	3.12 (2.39–4.53)	< 0.001
Fruits (servings/day)	1.56 (1.23–2.19)	2.37 (1.65–2.89)	3.41 (2.55–4.12)	< 0.001
Whole grains (servings/day)	1.27 (0.63–1.94)	2.19 (1.54–2.87)	3.39 (2.11–4.26)	< 0.001
Sugar‐sweetened beverages (servings/day)	0.17 (0.09–0.38)	0.12 (0.06–0.22)	0.08 (0.03–0.13)	< 0.001
Nuts and legumes (servings/day)	0.21 (0.12–0.35)	0.25 (0.14–0.42)	0.33 (0.19–0.53)	< 0.001
Red/processed meat (servings/day)	0.94 (0.61–1.44)	0.83 (0.54–1.32)	0.67 (0.38–1.25)	< 0.001
Trans fatty acids (% of energy)	2.24 (1.12–2.85)	1.68 (0.89–2.34)	0.63 (0.48–0.94)	< 0.001
Omega‐3 fatty acids (mg/day)	0.04 (0.02–0.09)	0.06 (0.04–0.13)	0.09 (0.07–0.16)	< 0.001
Polyunsaturated fatty acids (% of energy)	4.82 (3.85–6.13)	6.47 (4.55–8.21)	8.98 (5.32–10.25)	< 0.001
Sodium (mg/day)	3528 (2736–5476)	3317 (2583–5279)	3052 (2423–5067)	0.004

*Note:* Data are presented as mean ± standard deviation (SD) for normally distributed continuous variables. For non‐normally distributed variables (e.g., AHEI components), values are reported as median (interquartile range [IQR]). * in table 2 indicates *p* < 0.05 is significant.

Participants in the highest tertile of AHEI had significantly higher intakes of vitamin D (2.89 ± 1.22 μg/day vs. 1.83 ± 0.68 μg/day, *p* < 0.001), vitamin C (216.64 ± 93.46 mg/day vs. 156.32 ± 86.73 mg/day, *p* < 0.001), zinc, and calcium. Additionally, they consumed significantly higher amounts of vegetables, fruits, whole grains, nuts and legumes, and polyunsaturated fatty acids, while consuming lower amounts of trans fats, red meat, sodium, and sugar‐sweetened beverages (*p* < 0.05).

### 4.3 Biochemical and Clinical Variables Across PRAL Tertiles

The distribution of biochemical and clinical variables across PRAL tertiles is shown in Table 3. Participants in the highest tertile of PRAL had significantly higher scores for both the Fatty Liver Index and Hepatic Steatosis Index compared to the lowest tertile (*p* < 0.001). Participants in the third tertile also had significantly higher levels of ALT and GGT compared to the reference group. However, no significant differences were observed across PRAL tertiles for other anthropometric and biochemical variables.

**TABLE 3:** Distribution of laboratory and clinical variables across tertiles of the dietary inflammatory index (DII).

Variable	Tertile 1 (*n* = 140)	Tertile 2 (*n* = 140)	Tertile 3 (*n* = 140)	*p*
PRAL (mEq/day) (mean ± SD)	−52.39 (−34.8 to −19.28)	−45.11 (−26.3 to −10.26)	−41.79 (−63.86 to −8.79)	< 0.001
DAL (mEq/day) (mean ± SD)	10.34 ± 5.37	42.25 ± 6.73	58.49 ± 11.18	< 0.001
Body mass index (kg/m^2^)	26.74 ± 6.43	27.13 ± 5.82	27.55 ± 7.45	0.23
Waist circumference (cm)	93.72 ± 14.38	95.64 ± 15.69	95.73 ± 16.82	0.48
Fatty liver index	69.33 ± 10.36	75.64 ± 10.96	79.43 ± 12.13	< 0.001
Steatosis index	38.32 ± 5.26	43.52 ± 6.54	50.39 ± 6.37	< 0.001
TyG index	7.83 ± 1.80	7.87 ± 1.75	7.98 ± 1.82	0.53
Fasting blood sugar (mg/dL)	93.23 ± 16.39	90.84 ± 14.87	95.62 ± 16.47	0.31
Triglycerides (mg/dL)	158.23 ± 48.37	163.45 ± 57.26	165.24 ± 60.22	0.27
Total cholesterol (mg/dL)	176.51 ± 48.22	183.24 ± 53.27	187.20 ± 59.62	0.39
LDL cholesterol (mg/dL)	111.43 ± 24.19	109.52 ± 20.33	117.80 ± 28.76	0.22
HDL cholesterol (mg/dL)	48.65 ± 16.53	46.27 ± 17.88	41.27 ± 15.23	0.18
Alanine aminotransferase (mg/dL)	19.76 ± 5.43	23.45 ± 6.68	30.75 ± 8.23	0.002
Aspartate aminotransferase (mg/dL)	20.89 ± 5.72	22.49 ± 6.22	23.62 ± 7.64	0.26
Gamma‐glutamyl transferase (mg/dL)	21.02 ± 6.19	23.19 ± 5.90	28.47 ± 6.78	0.01

*Note:* Data are presented as mean ± standard deviation (SD) for continuous variables. For PRAL, values are reported as median (range) due to non‐normal distribution.

### 4.4 Association Between Dietary Indices and NAFLD Predictors

#### 4.4.1 AHEI and NAFLD Predictors

The associations between dietary indices and elevated levels of NAFLD predictors are presented in Table 4. Participants in the highest tertile of AHEI had a 69% lower likelihood of elevated FLI compared to the reference tertile in the crude model (OR = 0.31, 95% CI: 0.10–0.83, *p* = 0.025). FLI scores above 60 are considered a strong indicator of NAFLD in various studies. After adjustment for confounders, individuals in the third tertile of AHEI had a 62% lower probability of elevated FLI (OR = 0.38, 95% CI: 0.14–0.95, *p* = 0.030).

**TABLE 4:** Association between non‐alcoholic fatty liver disease (NAFLD) indices and dietary indices.

Variables	AHEI tertiles			*p* trend	PRAL tertiles			*p* trend	DAI tertiles			*p* trend
	1	2	3		1	2	3		1	2	3	
**FLI (> 60)**												
**Crude model**	Reference	0.89 (0.37, 1.67)	0.31 (0.10, 0.83)	**0.025**	Reference	1.36 (0.87, 2.64)	2.28 (1.10, 3.45)	**0.037**	Reference	0.91 (0.36, 1.83)	0.56 (0.21, 1.45)	0.224
**Adjusted model**	Reference	0.95 (0.45, 1.86)	0.38 (0.14, 0.95)	**0.030**	Reference	1.16 (0.78, 2.35)	1.98 (1.02, 3.22)	**0.042**	Reference	1.05 (0.43, 1.92)	0.62 (0.30, 1.50)	0.38
**HSI (> 36)**												
**Crude model**	Reference	0.97 (0.54, 1.85)	0.35 (0.21, 0.71)	**0.002**	Reference	1.58 (0.75, 2.92)	2.51 (1.30, 3.80)	**< 0.001**	Reference	0.67 (0.31, 1.41)	0.40 (0.19, 0.86)	**0.001**
**Adjusted model**	Reference	1.13 (0.67, 2.12)	0.40 (0.26, 0.75)	**0.005**	Reference	1.44 (0.77, 2.75)	2.48 (1.31, 3.55)	**0.002**	Reference	0.65 (0.35, 1.38)	0.41 (0.22, 0.89)	**0.005**
**TyG index (> 7)**												
**Crude model**	Reference	1.24 (0.66, 2.35)	0.72 (0.45, 1.45)	0.26	Reference	1.44 (0.71, 2.13)	1.89 (1.12, 3.26)	**< 0.001**	Reference	0.77 (0.46, 1.69)	0.32 (0.11, 0.90)	**< 0.001**
**Adjusted model**	Reference	1.18 (0.68, 2.32)	0.75 (0.46, 1.35)	0.42	Reference	1.36 (0.75, 2.10)	1.80 (1.08, 3.23)	**0.001**	Reference	0.81 (0.50, 1.61)	0.36 (0.13, 0.96)	**< 0.001**

*Note:* Calculated using logistic regression. The adjusted model accounts for age, physical activity, gender, social status, body mass index, and energy intake. Bold values in table 4 indicates, *p* < 0.05 is significant.

Regarding HSI, participants in the highest tertile of AHEI were 65% less likely to have elevated HSI scores in the crude model (OR = 0.35, 95% CI: 0.21–0.71, *p* = 0.002) and 60% less likely in the adjusted model (OR = 0.40, 95% CI: 0.26–0.75, *p* = 0.005). No significant association was found between AHEI tertiles and elevated TyG index in either the crude (*p* = 0.26) or adjusted models (*p* = 0.42).

#### 4.4.2 PRAL and NAFLD Predictors

Participants in the highest tertile of PRAL had a 1.98 times higher likelihood of elevated FLI compared to the reference group (OR = 1.98, 95% CI: 1.02–3.22, *p* = 0.042) in the adjusted model. Similarly, those in the highest tertile of PRAL were 2.48 times more likely to have elevated HSI (OR = 2.48, 95% CI: 1.31–3.55, *p* = 0.002) and 1.80 times more likely to have elevated TyG index (OR = 1.80, 95% CI: 1.08–3.23, *p* = 0.001).

#### 4.4.3 DAI and NAFLD Predictors

No significant association was observed between DAI tertiles and elevated FLI in either the crude (*p* = 0.224) or adjusted models (*p* = 0.38). However, participants in the third tertile of DAI had 59% lower odds of elevated HSI and 64% lower odds of elevated TyG in the adjusted model.

## 5 Discussion

This cross‐sectional study revealed that higher diet quality, as assessed by the AHEI, was associated with lower levels of FLI, HSI, triglycerides (TG), gamma‐glutamyl transferase (GGT), and alanine aminotransferase (ALT), as well as reduced intake of trans fats, red meat, sodium, and sugar‐sweetened beverages. Additionally, higher AHEI scores were linked to increased levels of HDL cholesterol and greater consumption of vitamin C, vitamin D, zinc, calcium, vegetables, fruits, whole grains, nuts and legumes, polyunsaturated fatty acids (PUFA), and omega‐3 fatty acids. Conversely, higher PRAL was associated with increased FLI, ALT, and GGT levels, along with a higher TyG index. For the DAI, higher scores were linked to lower HSI and TyG levels.

### 5.1 Diet Quality Index (AHEI)

Our findings are consistent with previous studies demonstrating an inverse relationship between AHEI and metabolic risk factors such as BMI, waist circumference, fasting blood glucose, and blood pressure (Al Kudsee et al. 2022).

The AHEI emphasizes the intake of nutrient‐dense foods such as vegetables, fruits, whole grains, nuts, and unsaturated fats while limiting red and processed meats, trans fats, sodium, and sugar‐sweetened beverages (Zhang et al. 2024). The observed benefits of higher AHEI adherence can be attributed to several mechanisms. For instance, dietary fiber from fruits, vegetables, and whole grains plays a key role in reducing cholesterol absorption, improving insulin sensitivity, and regulating glucose metabolism. Polyphenols and carotenoids, abundant in plant‐based foods, exhibit potent antioxidant and anti‐inflammatory properties, reducing oxidative stress and mitigating liver inflammation (Bohn et al. 2022).

Additionally, unsaturated fats, particularly polyunsaturated fatty acids (PUFAs) and omega‐3 fatty acids, are known to improve lipid profiles by reducing triglyceride levels and increasing HDL cholesterol. They also enhance endothelial function and reduce the risk of atherosclerosis, which is closely linked to metabolic disturbances. The cumulative effect of these dietary components underscores the importance of adhering to a high‐quality diet in mitigating metabolic dysfunction and reducing NAFLD risk (Hamamah et al. 2024; Park et al. 2020; Zhang et al. 2020).

In agreement with our findings, large‐scale studies, including cross‐sectional analyses and cohort studies, have demonstrated that higher adherence to the AHEI is associated with decreased odds of MetS, reduced waist circumference, improved HDL cholesterol levels, and lower risks of hyperglycemia and hypertriglyceridemia (Al Kudsee et al. 2022; Fallaize et al. 2018; Mattei et al. 2016; Saraf‐Bank et al. 2017). By focusing on the overall dietary pattern rather than individual nutrients, the AHEI captures the synergistic effects of dietary components, offering a comprehensive approach to dietary assessment and its relationship with health outcomes (Godoy‐Izquierdo et al. 2021).

Building on the protective role of high‐quality diets, our results also indicate associations between higher PRAL scores and adverse liver health outcomes, including increased FLI, HSI, and TyG index, as well as elevated levels of ALT and GGT. PRAL reflects the net acid load of the diet, with higher scores indicating diets rich in acid‐forming foods such as red and processed meats, dairy products, and refined grains, and lower consumption of alkaline‐forming foods like fruits and vegetables. We selected PRAL over other DAL measures like Net Endogenous Acid Production (NEAP) because PRAL provides a more comprehensive estimate by incorporating additional minerals (e.g., phosphorus, calcium, magnesium) beyond just protein and potassium, allowing better capture of dietary nuances in metabolic studies (Cheng and Wang 2023; Jahromi et al. 2023).

Mechanistically, a high DAL is associated with chronic low‐grade metabolic acidosis, disrupting the body's acid–base balance (Remer and Manz 1995). This acidic environment has been shown to be linked with insulin resistance, a key driver of NAFLD, by impairing glucolipid metabolism and enhancing hepatic lipogenesis (Marušić et al. 2021). Insulin resistance also may contribute to the activation of hepatic stellate cells and hepatocyte damage, potentially exacerbating the progression of liver fibrosis and steatosis.

Moreover, chronic metabolic acidosis is associated with impairment of the growth hormone‐insulin‐like growth factor‐1 (GH‐IGF‐1) axis, further exacerbating metabolic dysfunction and increasing susceptibility to NAFLD (Dichtel et al. 2022; Green and Maor 2000). The reduced buffering capacity resulting from inadequate consumption of base‐forming foods (e.g., fruits and vegetables) further aggravates this imbalance.

Several studies have corroborated our findings. For example, several researchers have demonstrated that PRAL is positively associated with surrogate markers of NAFLD, including FLI and HSI, and elevated liver enzymes such as ALT (Alferink et al. 2019; Krupp et al. 2012). Additionally, diets high in animal protein, a major contributor to DAL, have been linked to higher risks of insulin resistance, NAFLD, and liver‐related mortality (Alferink et al. 2019). The detrimental effects of an acid‐forming diet emphasize the need for dietary interventions that promote the consumption of alkaline foods to restore acid–base balance and reduce the burden of NAFLD.

Shifting focus to antioxidants, our study also highlights associations between higher DAI scores and lower HSI and TyG indices, suggesting potential protective effects against metabolic and liver‐related abnormalities. Notably, no significant association was found with FLI, which may reflect differential sensitivities among NAFLD indices: FLI incorporates anthropometric measures (e.g., BMI, waist circumference) that could be less directly influenced by dietary antioxidants, whereas HSI (enzyme‐based) and TyG (triglyceride‐glucose product) may be more responsive to oxidative stress modulation in liver function and insulin resistance pathways (Bakhshi et al. 2025; Emamat et al. 2023).

Antioxidants, including vitamins A, C, and E, as well as minerals such as selenium and zinc, play a crucial role in neutralizing free radicals and oxidative stress, which are major contributors to liver damage and metabolic dysfunction (Ferramosca et al. 2017).

The liver, as a central organ in the detoxification process, is particularly vulnerable to oxidative stress caused by the accumulation of reactive oxygen species (ROS) (Lobo et al. 2010). Oxidative stress disrupts hepatocyte function, promotes inflammation, and accelerates the progression of liver diseases such as NAFLD (Muriel 2009). The synergistic action of dietary antioxidants, including enzymatic and non‐enzymatic components, helps maintain redox balance and protect liver cells from oxidative damage (Lobo et al. 2010).

Mechanistically, antioxidants improve lipid metabolism by modulating the expression and activity of proteins involved in lipid transport and storage (Ferramosca et al. 2017). They also reduce inflammation by suppressing pro‐inflammatory cytokines and enhancing the activity of anti‐inflammatory pathways (Vahid et al. 2021). Diets rich in antioxidant‐containing foods, such as fruits, vegetables, nuts, and seeds, provide a diverse array of bioactive compounds that work synergistically to support liver health.

Although limited research has specifically examined the relationship between DAI and NAFLD, studies on antioxidant‐rich dietary patterns, such as the Mediterranean diet, have demonstrated significant protective effects against metabolic syndrome and liver‐related disorders (Chen et al. 2016). On the other hand, in line with our findings, another study showed that there is a significant relationship between DAI and metabolic syndrome (Abdollahpour et al. 2022). The findings of this study align with previous evidence, suggesting that dietary antioxidants represent a promising strategy for preventing and managing NAFLD.

Contextualizing these results, our findings in an Iranian cohort may differ from those in Western populations due to dietary variations. For instance, Iranian diets often feature higher dairy and grain consumption, which can elevate DAL through acid‐forming components, potentially amplifying associations with NAFLD compared to Western diets high in processed meats and sugars but lower in certain dairy staples (Bakhshi et al. 2025). However, both patterns generally contribute to higher DAL, underscoring the need for cross‐cultural comparisons.

This study has several strengths, including a comprehensive evaluation of dietary patterns using three well‐established indices: AHEI, PRAL, and DAI. This multifaceted approach allowed us to capture various aspects of diet quality, acid–base balance, and antioxidant capacity, offering a holistic view of their associations with liver enzymes, lipid profiles, and NAFLD predictors in Iranian MetS patients. The use of validated dietary assessment tools and biochemical markers enhances the reliability and accuracy of the findings. This study addresses an important public health issue in a high‐risk population, providing insights that may inform targeted dietary interventions.

However, this study also has some limitations. Its cross‐sectional design prevents the establishment of causality between dietary indices and NAFLD‐related outcomes. Additionally, the use of self‐reported dietary data through a food frequency questionnaire (FFQ) may be subject to recall bias or underreporting, particularly for unhealthy food items. Another limitation is the potential influence of unmeasured confounders, such as genetic predisposition, physical activity patterns, or other lifestyle factors, which could affect the observed associations. Lastly, the findings are based on a specific population—Iranian population with MetS, limiting the generalizability of the results to other demographic or ethnic groups. Future longitudinal and interventional studies are needed to confirm these associations and establish causal relationships.

## 6 Conclusion

In this cross‐sectional study of 420 patients with MetS, we observed significant associations between dietary indices and markers of cardiometabolic health and NAFLD risk. Higher scores on the AHEI and DAI were linked to improved lipid profiles, lower liver enzyme levels (e.g., ALT and GGT), and reduced NAFLD predictive indices (e.g., FLI and HSI). Conversely, higher DAL was associated with elevated FLI and ALT, suggesting a detrimental impact on liver health. These findings underscore the potential role of high‐quality, antioxidant‐rich, and acid–base balanced diets in mitigating NAFLD risk among individuals with MetS.

Our results align with emerging evidence that dietary patterns can modulate metabolic dysregulation and oxidative stress, key drivers of NAFLD progression in MetS patients. Clinically, these insights support dietary interventions emphasizing fruits, vegetables, whole grains, and antioxidants while minimizing acidogenic foods like processed meats and refined grains.

Looking ahead, future research should prioritize longitudinal studies to establish causality between these dietary indices and NAFLD outcomes, as the cross‐sectional design limits inferences about temporality. Interventional trials testing targeted dietary modifications could validate these associations and inform personalized nutrition strategies. Additionally, exploring underlying mechanisms—such as gut microbiota modulation or inflammatory pathways—and extending investigations to diverse ethnic and socioeconomic populations would enhance the generalizability and translational potential of these findings.

## Author Contributions

M.R. conceived the study, B.G., S.A.S., F.M.S., V.N., and M.R. collected and analyzed the data. M.R. interpreted the statistical analyses and V.N. wrote the first draft of the manuscript. M.R. contributed to the manuscript revising and editing. All of the authors critically revised the manuscript. The authors read and approved the final manuscript.

## Ethics Statement

This study was approved by the Ethics Committee of Zanjan University of Medical Sciences (approval code: [IR.ZUMS.REC.1403.004]). Written informed consent was obtained from all participants after explaining the study objectives. All patients were provided with written and verbal information about the study, and informed consent was obtained from all subjects.

## Consent

The authors have nothing to report.

## Conflicts of Interest

The authors declare no conflicts of interest.

## Data Availability

The data and all supporting materials used in the preparation of this manuscript are freely available from the corresponding author at reasonable request.
